# The Cytolethal Distending Toxin Produced by Nontyphoidal *Salmonella* Serotypes Javiana, Montevideo, Oranienburg, and Mississippi Induces DNA Damage in a Manner Similar to That of Serotype Typhi

**DOI:** 10.1128/mBio.02109-16

**Published:** 2016-12-20

**Authors:** Rachel A. Miller, Martin Wiedmann

**Affiliations:** Department of Food Science, Cornell University, Ithaca, New York, USA

## Abstract

Select nontyphoidal *Salmonella enterica* (NTS) serotypes were recently found to encode the *Salmonella* cytolethal distending toxin (S-CDT), an important virulence factor for serotype Typhi, the causative agent of typhoid fever. Using a PCR-based assay, we determined that among 21 NTS serotypes causing the majority of food-borne salmonellosis cases in the United States, genes encoding S-CDT are conserved in isolates representing serotypes Javiana, Montevideo, and Oranienburg but that among serotype Mississippi isolates, the presence of S-CDT-encoding genes is clade associated. HeLa cells infected with representative strains of these S-CDT-positive serotypes had a significantly higher proportion of cells arrested in the G_2_/M phase than HeLa cells infected with representative strains of S-CDT-negative serotypes Typhimurium, Newport, and Enteritidis. The G_2_/M cell cycle arrest was dependent on CdtB, the active subunit of S-CDT, as infection with isogenic Δ*cdtB* mutants abolished their ability to induce a G_2_/M cell cycle arrest. Infection with S-CDT-encoding serotypes was significantly associated with activation of the host cell’s DNA damage response (DDR), a signaling cascade that is important for detecting and repairing damaged DNA. HeLa cell populations infected with S-CDT-positive serotypes had a significantly higher proportion of cells with DDR protein 53BP1 and γH2AX foci than cells infected with either S-CDT-negative serotypes or isogenic Δ*cdtB* strains. Intoxication with S-CDT occurred via autocrine and paracrine pathways, as uninfected HeLa cells among populations of infected cells also had an activated DDR. Overall, we show that S-CDT plays a significant role in the cellular outcome of infection with NTS serotypes.

## IMPORTANCE

The recent discovery that multiple serotypes encode S-CDT, which was previously established as an important virulence factor for serotype Typhi, suggested that this toxin may also contribute to the outcome of infection with nontyphoidal serotypes. In this study, we demonstrate that at a cellular level, S-CDT significantly alters the outcome of infection by inducing DNA damage which is associated with a cell cycle arrest and activation of the host cell’s DDR. Importantly, these results contribute valuable information for assessing the public health implications of S-CDT in infections with NTS serotypes. Our data suggest that infection with *Salmonella* strains that encode S-CDT has the potential to result in DNA damage, which may contribute to long-term sequelae.

## INTRODUCTION

Cytolethal distending toxins (CDTs) are important virulence factors produced by Gram-negative bacteria, including those causing predominantly extracellular infections (*Aggregatibacter actinomycetemcomitans*, *Haemophilus* spp., *Providencia alcalifaciens*) and those able to cause intracellular infections (*Campylobacter* spp., *Escherichia coli*, *Helicobacter* spp., *Salmonella enterica*, *Shigella* spp., and *Yersinia* spp.) (1, 2). *In vitro*, CDTs act as genotoxins, inducing single-strand or double-strand breaks, resulting in activation of the eukaryotic cell DNA damage response (DDR) (3–5). DDR protein 53 binding protein 1 (53BP1) and phosphorylation of histone 2AX (producing γH2AX) have been shown previously to localize at DNA double-strand breaks, recruiting DNA repair machinery to the DNA damage (6, 7). *In vivo*, CDT-induced damage has been associated with enhanced colonization of the host by CDT-producing pathogens, tumorigenesis, and neoplastic lesions, and to contribute to the establishment of chronic infections (8–10).

The CDTs encoded by most Gram-negative pathogens exist as an AB_2_ toxin composed of 2 binding subunits, CdtA and CdtC, and an active subunit, CdtB (3). *In vitro* analyses have demonstrated nuclease activity of the CdtB subunit using plasmid relaxation assays (11). However, the CDT encoded by select *S. enterica* serotypes (referred to as S-CDT, for *Salmonella* cytolethal distending toxin) represents a unique form of CDT with an A_2_B_5_ configuration with 2 active subunits (CdtB and PltA) and 5 binding subunits (PltB) arranged as a pentameric ring (2, 12). The PltA subunit shares structural and functional homology with the S1 subunit of the pertussis toxin, which acts as an ADP-ribosyl transferase (2, 13). The PltB subunit has been suggested to play a role in the binding of S-CDT to host cell receptors, as it shares homology with the binding subunits of both the pertussis toxin (subunits S2 and S3) and the subtilase cytotoxin (SubB) produced by *E. coli* (12–14).

S-CDT was originally characterized as a unique virulence factor of *S. enterica* subspecies *enterica* serotype Typhi (2, 15, 16). *In vitro* studies demonstrated that, like CDTs produced by other Gram-negative pathogens, the S-CDT produced by S. Typhi induced DNA damage leading to a G_2_/M arrest among infected cell culture populations (2, 12, 16). Recent studies suggest that the S-CDT produced by S. Typhi is capable of recapitulating symptoms of typhoid fever, modulating the immune system, and enabling S. Typhi to persist *in vivo* (8, 12).

Each year, nontyphoidal *S. enterica* (NTS) infections account for approximately 1.03 million cases of food-borne illness in the United States and an estimated 80.3 million cases internationally (17, 18). There are marked differences in disease severity resulting from infections with different *Salmonella* serotypes (19), yet genetic analyses have failed to definitively account for differences in virulence among NTS serotypes (20, 21). Recent studies have identified genes encoding S-CDT in >40 NTS serotypes (20, 22). However, little is known regarding the distribution and conservation of S-CDT-encoding genes among NTS serotypes and the contributions of S-CDT in the course of infection with NTS serotypes (23–25).

In this study, we examined differences in the cellular outcomes of infection between S-CDT-positive and -negative NTS serotypes. Using a PCR-based screen, we characterized the distribution and sequence conservation of S-CDT-encoding genes among 21 of the NTS serotypes most frequently isolated from clinical cases of salmonellosis in the United States. Next, we examined the cellular response to infection with the 4 S-CDT-positive serotypes identified through our PCR screen and compared this to the responses to infection with 3 S-CDT-negative serotypes in order to determine what role S-CDT may play in a cell model of infection. Overall, we show that genes encoding S-CDT are highly conserved among isolates representing NTS serotypes Javiana, Montevideo, and Oranienburg, as well as among isolates of 1 clade of serotype Mississippi. Importantly, infection with NTS serotypes encoding S-CDT significantly alters the outcome of infection in a HeLa cell model. These data suggest that S-CDT is an important virulence factor in NTS strains, which may contribute to differences in disease severity observed among different NTS serotypes. With the recognized potential of CDTs to serve as genotoxins, our data also suggest that infection with some *S. enterica* strains (i.e., those that carry S-CDT) has the potential to cause DNA damage in host cells, which may predispose case patients to long-term sequelae that remain to be defined.

## RESULTS

### Genes encoding S-CDT are conserved among isolates of NTS serotypes Javiana, Montevideo, and Oranienburg, while for serotype Mississippi isolates, genes encoding S-CDT are clade associated.

**FIG 1:**
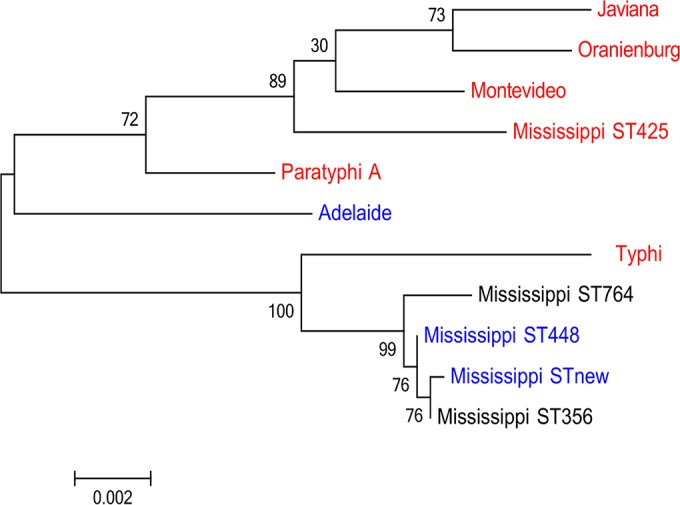
Genes encoding S-CDT are clade restricted for serotype Mississippi. Maximum-likelihood tree showing phylogenetic clades based on concatenated 7-gene MLST data for serotype Mississippi, closely related serotypes Adelaide, Paratyphi A, and Typhi (22), and S-CDT-positive serotypes Javiana, Oranienburg, and Montevideo, which are included for comparison. MLST data for the Mississippi isolates designated STnew, ST356, ST425, ST448, and ST764 were obtained for this study; all other sequence data were extracted from the MLST database (26). Branch lengths represent a substitution rate of 2 nucleotides per 1,000. Numbers at nodes represent bootstrap values based on 1,000 repetitions. Blue represents S-CDT-negative MLSTs and serotypes as determined in this study and by den Bakker et al. (22); red represents S-CDT-positive MLSTs and serotypes. The S-CDT status for serotype Mississippi ST764 and ST356 are unknown, and these STs are thus shown in black lettering.

A PCR screen used to detect internal gene fragments for *pltA*, *pltB*, and *cdtB* showed that all isolates of *S. enterica* serotypes Javiana, Montevideo, and Oranienburg screened (50 isolates for each serotype), which are among 21 of the NTS serotypes most frequently isolated from clinical cases of salmonellosis in the United States, encoded all 3 toxin components. For serotype Mississippi, only 4 of the 8 isolates tested were S-CDT positive (Table 1). Subsequent 7-gene multilocus sequence type (MLST) characterization of the 8 serotype Mississippi isolates indicated that this serotype is polyphyletic and that these isolates represent 2 distinct clades (Fig. 1). S-CDT-encoding genes were present in all 4 isolates with sequence type 425 (ST425) (Fig. 1) (26), while the 4 isolates in the other clade (representing ST448 and a new ST) were negative for the S-CDT genes. Two other *Salmonella* serotype Mississippi STs (i.e., ST356 and ST764) obtained from the MLST database also clustered into the S-CDT-negative clade (26). Interestingly, isolates of the S-CDT-negative Mississippi clade clustered with S. Typhi (S-CDT positive), suggesting that this clade may have lost the S-CDT genes (Fig. 1).

**TABLE 1:** Distribution of S-CDT-encoding genes among 21 nontyphoidal *Salmonella enterica* serotypes most commonly isolated from human cases in the United States in 2011

Serotype	Serogroup	No. of reported laboratory-confirmed human clinical cases in 2011^a^	Proportion of S-CDT-positive isolates^b^	
			%	No. positive/total
Enteritidis	D_1_	7,553	0	0/50
Typhimurium^c^	B	6,131	0	0/50
Newport	C_2_−C_3_	5,211	0	0/50
Javiana	D_1_	2,937	100	50/50
I 4, [5], 12:i: −	B	1,339	0	0/50
Montevideo	C_1_	1,196	100	50/50
Heidelberg	B	1,103	0	0/50
München	C_2_−C_3_	984	0	0/48
Infantis	C_1_	910	0	0/50
Braenderup	C_1_	739	0	0/50
Oranienburg	C_1_	721	100	50/50
Saint Paul	B	709	0	0/50
Mississippi	G	549	50	4/8
Thompson	C_1_	536	0	0/50
Agona	B	505	0	0/50
Bareilly	C_1_	429	0	0/22
Berta	D_1_	321	0	0/20
Anatum	E_1_	282	0	0/50
Hartford	C_1_	241	0	0/18
I 13,23:b: −	G	218	0	0/1
Hadar	C_2_−C_3_	205	0	0/47

a Reported laboratory-confirmed human clinical cases in 2011 according to the CDC’s National *Salmonella* Surveillance report of 2011.

b The presence of all S-CDT components (*pltA*, *pltB*, and *cdtB*) as determined by PCR amplification and the proportion of S-CDT-positive strains tested per serotype.

c Serotype Typhimurium includes Typhimurium variant O:5−.

Based on CDC data on the frequency of different NTS serotypes among human clinical cases in the United States, strains representing the 3 serotypes Javiana, Montevideo, and Oranienburg (among which all isolates were S-CDT positive) were the cause of 4,854 of the 32,819 reported cases of nontyphoidal salmonellosis caused by the 21 NTS serotypes screened (listed in Table 1). Serotype Mississippi, on the other hand, accounted for 549 reported cases. With 4/8 serotype Mississippi isolates testing as S-CDT positive, we can estimate with a 90% confidence interval (CI) of 0.25 to 0.75 that the binomial proportion of human Mississippi isolates positive for S-CDT is 0.5. Based on this, infections with S-CDT-encoding Mississippi strains account for approximately 275 cases per year (90% CI, 137 to 412). Overall, S-CDT-positive isolates among these 21 human clinical NTS-associated serotypes were estimated to cause approximately 5,129 reported human cases in the United States. Because Scallan et al. (17) previously suggested that only 1 out of each 29.3 cases of nontyphoidal salmonellosis is reported (90% CI, 21.8 to 38.4), we estimated that approximately 150,280 cases (90% CI, 111,812 to 196,954) of salmonellosis in the United States are caused by S-CDT-positive isolates representing serotypes Javiana, Montevideo, Oranienburg, and Mississippi.

### *pltA*, *pltB*, and *cdtB* are highly conserved among NTS serotypes encoding S-CDT.

To characterize the sequence conservation of *pltA*, *pltB*, and *cdtB* (encoding S-CDT subunits PltA, PltB, and CdtB, respectively), we sequenced PCR amplicons for each of these genes for 5 representative isolates from each of the S-CDT-positive serotypes, except for serotype Mississippi, for which all 4 S-CDT-positive isolates were characterized. Overall, the predicted peptide products of *pltA*, *pltB*, and *cdtB* were highly conserved across serotypes (including in S. Typhi strain CT-18), with 98.3%, 94.9%, and 99.3% of amino acids conserved, respectively. DNA sequences for *pltA*, *pltB*, and *cdtB* were also highly conserved, with 98.1%, 96.1%, and 99.4% of nucleotides being identical among all isolates, respectively.

The CdtB subunit was the most conserved among the toxin-encoding subunits. Among the isolates analyzed, we found 2 CdtB sequence types. All serotype Javiana, Montevideo, and Oranienburg isolates had the same CdtB amino acid sequences (see Table 2), which were identical to that of *S.* Typhi strain CT-18 (designated CdtB sequence type 1). CdtB sequence type 2 was found in all S-CDT-positive serotype Mississippi isolates, which had amino acid substitutions at residues 217 (Thr versus Ala in CT-18) and 223 (Ala versus Glu in CT-18). The cysteine residue (Cys269) essential for anchoring CdtB and PltA (12) was conserved among the predicted CdtB peptide sequences for all S-CDT-positive NTS isolates analyzed.

**TABLE 2:** Amino acid differences in S-CDT subunits between serotype Typhi and nontyphoidal serotypes

S-CDT subunit	Amino acid residue	Amino acid (nucleotide codon) in^a^:		Serotype(s) with substitution
		CT-18	Serotype(s)	
CdtB	217	Ala (**G**CC)	Thr (**A**CC)	Mississippi
	223	Glu (G**A**A)	Ala (G**C**A)	Mississippi
PltB^b^	3	Met (AT**G**)	Ile (AT**A**)	Javiana, Montevideo, Mississippi, Oranienburg
	4	Ser (A**G**T)	Asn (A**A**T)	Javiana, Montevideo, Mississippi, Oranienburg
	6	Tyr (T**A**T)	Phe (T**T**T)	Javiana, Montevideo, Mississippi, Oranienburg
	29	Asn (AA**T**)	Lys (AA**A**)	Javiana, Montevideo, Oranienburg
	50	Ser (**A**GT)	Gly (**G**GT)	Javiana, Oranienburg
	65	Thr (A**C**A)	Ile (A**T**A)	Javiana, Oranienburg (1 of the 5 isolates)
	116	Thr (**A**CC)	Ala (**G**CC)	Oranienburg (4 of the 5 isolates)
PltA^c^	6	Phe (**T**TC)	Leu (**C**TC)	Javiana, Oranienburg
	169	Ala (**G**CC)	Ser (**T**CC)	Javiana, Montevideo, Oranienburg
	215	His (CA**T**)	Gln (CA**A**)	Javiana, Montevideo, Mississippi, Oranienburg
	227	Met (**A**TG)	Val (**G**TG)	Javiana

a The three-letter abbreviations for the amino acid residues in *S. enterica* serotype Typhi strain CT-18 or in the nontyphoidal serotypes are shown; codons encoding the amino acid residues are shown in parentheses. The specific SNPs causing the amino acid change are shown in bold font.

b *Salmonella* Oranienburg strain FSL A4-0642 showed the same amino acid substitution in PltB as the 5 Javiana isolates tested.

c In addition to having the amino acid differences listed here, isolate FSL R8-1549 (serotype Mississippi) had an SNP resulting in an amino acid change from glycine to serine at position 211; this genotype was not detected in any other isolates.

The key amino acid residues for PltA, the ribosyl transferase subunit, were also conserved. Both the PltA amino acid residue implicated in binding to the CdtB subunit (Cys214) and the catalytic residue (Glu133) were conserved among all isolates. A total of 4 amino acid substitutions were identified as differing from the sequence of *S.* Typhi CT-18 (Table 2). Serotype-specific amino acid substitutions included (i) a His215 residue in *S.* Typhi strain CT-18 (compared to Gln215 for all other serotypes) and (ii) a Val227 residue in all Javiana isolates (compared to Met227 for all other isolates) (Table 2).

The PltB subunit was the most varied of the toxin subunits, with 7 variable amino acid residues (Table 2). At 3 variable sites (residues 3, 4, and 29), the PltB sequence for S. Typhi CT-18 differed from those of all other isolates. Other residues showed different amino acid substitution patterns. For example, all serotype Javiana isolates and 1 serotype Oranienburg isolate had Ile65 (in place of Thr65 in all serotype Montevideo isolates, the 4 remaining serotype Oranienburg isolates, and *S.* Typhi strain CT-18). For 1 Oranienburg isolate, the complete PltB amino acid sequence was identical to that of the Javiana isolates. We confirmed that this was not due to misidentification of this isolate, as the 7-gene MLST for this isolate matched STs of other serotype Oranienburg isolates. All key residues involved in binding to sugar moieties (Tyr33, Ser35, and Lys59) were conserved across all serotypes (12).

### Infection with S-CDT-positive serotypes results in G_2_/M cell cycle arrest, while infection with S-CDT-negative serotypes does not.

**FIG 2:**
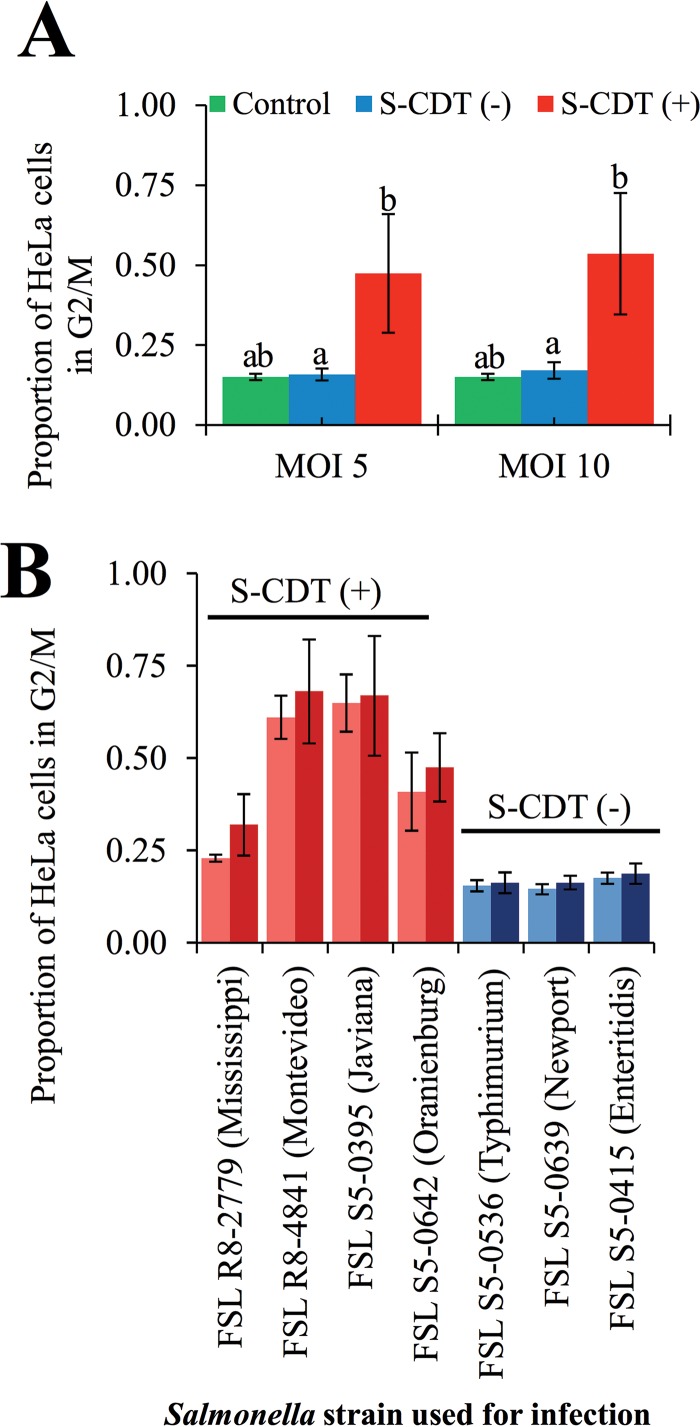
Infection with NTS serotypes encoding S-CDT results in significantly higher proportions of cells arrested in G_2_/M in HeLa cells than in uninfected cells. HeLa cells were infected with isolates representing S-CDT-positive serotypes (Mississippi, Montevideo, Javiana, and Oranienburg) and S-CDT-negative serotypes (Enteritidis, Typhimurium, and Newport) at multiplicities of infection (MOIs) of approximately 5 and 10. At 48 hpi, cells were fixed and stained with PI. DNA content was analyzed by flow cytometry, and cells were gated to exclude multiplets. Least-square means from a linear mixed-effects model were used to determine significant differences in proportions of HeLa cells in the G_2_/M phase for cells infected with the 2 MOIs. Results represent 4 independent experiments. (A) Bar chart showing average proportions of cell populations in G_2_/M phase among HeLa cells infected with S-CDT-negative (blue bars) or S-CDT-positive (red bars) serotypes at 2 MOIs (5 and 10, shown on the *x* axis); data shown are the averages for the 4 S-CDT-positive and the 3 S-CDT-negative strains. Green bars represent values for uninfected control cells (4 independent replicates). Bars that do not have identical letters represent statistically significantly different proportions of cells in G_2_/M phase (*P* < 0.05). (B) Bar chart showing G_2_/M arrest resulting from infection with the individual *Salmonella* strains used in this study (Table 3). Light-blue and red bars represent infection at an MOI of 5, and dark-blue and dark-red bars represent infection at an MOI of 10. Statistical models are based on log-transformed data; nontransformed data are shown. Error bars represent standard deviations.

To determine whether the presence of S-CDT-encoding genes altered the outcome of infection at the cellular level, we infected HeLa cells with *S. enterica* isolates representing S-CDT-positive and S-CDT-negative serotypes. Overall, we found that HeLa cell populations infected with isolates representing S-CDT-positive serotypes (i.e., Javiana, Montevideo, Mississippi, and Oranienburg) showed a significantly higher proportion of cells in the G_2_/M phase (Fig. 2A and B) than HeLa cells infected with S-CDT-negative serotypes (i.e., Enteritidis, Newport, and Typhimurium), both at a multiplicity of infection (MOI) of 5 (*P* = 0.016) and at an MOI of 10 (*P* = 0.011). For example, for an MOI of 10, the proportion of HeLa cells in the G_2_/M phase ranged from 25.9% to 89.7% for the different S-CDT-positive isolates and from 13.8% to 21.1% for the S-CDT-negative isolates (Fig. 2A). For HeLa cells infected with S-CDT-negative serotypes, the proportion of cells in the G_2_/M phase did not significantly differ from the proportion of uninfected cells in the G_2_/M phase, regardless of the MOI (MOI 5, *P* = 0.999; MOI 10, *P* = 0.998). We also found a significant effect (*P* < 0.0001) of the isolate used for infection on the proportion of HeLa cells in G_2_/M phase. Tukey’s *post hoc* test specifically showed significant differences in the magnitudes of G_2_/M arrest caused by the different S-CDT-positive isolates tested (Fig. 2); HeLa cells infected with either serotype Mississippi or serotype Oranienburg strains showed a significantly lower (*P* < 0.05) proportion of HeLa cells in the G_2_/M phase than HeLa cells infected with either serotype Javiana or serotype Montevideo isolates. Importantly, even for infections with Mississippi and Oranienburg isolates, the proportion of HeLa cells in the G_2_/M phase was still significantly (*P* < 0.05) higher than for any of the infections involving S-CDT-negative isolates. Overall, our results indicate that infection with S-CDT-encoding serotypes is associated with a significant increase in the proportion of cell populations arrested in the G_2_/M phase, indicative of DNA damage.

### S-CDT-encoding serotypes activate the DNA damage response, while S-CDT-negative or S-CDT-null serotypes do not.

**FIG 3:**
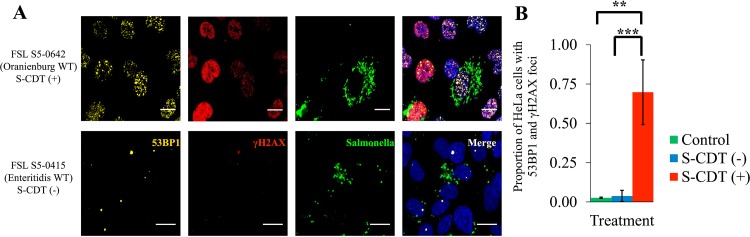
Infection with S-CDT-encoding serotypes is associated with activation of the DNA damage response. HeLa cells grown on glass coverslips were infected with *Salmonella* strains at a multiplicity of infection of approximately 5. Cells were fixed at 48 hpi and were stained with antibodies recognizing 53BP1, γH2AX, and *Salmonella* cells. DAPI was included as a nucleic acid stain. (A) Representative images for strains FSL S5-0642 (serotype Oranienburg; S-CDT positive) and FSL S5-0415 (serotype Enteritidis; S-CDT negative); data for other strains are shown in Fig. S1 in the supplemental material. Scale bars, 25 μm. (B) A mixed-effects linear model was used to determine whether the proportions of HeLa cells that were positive for 53BP1 and γH2AX foci at 48 hpi differed between S-CDT-positive and -negative serotypes and uninfected controls. Data represent 2 technical replicates from 2 independent experiments for each serotype. The S-CDT-negative bar represents the average of all data for HeLa cells infected with serotypes Typhimurium, Newport, and Enteritidis; the S-CDT-positive bar represents the average for all data for HeLa cells infected with serotypes Javiana, Montevideo, Mississippi, and Oranienburg. ***, *P* < 0.001; **, *P* < 0.01. Although the data were transformed for statistical analyses, nontransformed data are shown. Bars represent standard deviations.

CdtB, the active component of S-CDT has nuclease activity *in vitro* and activates the host cell’s DDR (3, 12, 16). To determine whether infection with NTS serotypes encoding S-CDT resulted in an activated DDR among infected HeLa cell populations, we used immunofluorescence staining to detect 53BP1 foci and then confirmed this by also staining for phosphorylated serine 139 on H2AX (yielding γH2AX) (27, 28). HeLa cell populations infected with S-CDT-encoding *Salmonella* had a significantly higher proportion of cell populations with both 53BP1 and γH2AX foci (“double-positive cells”) (Fig. 3A and B) than either HeLa cells infected with S-CDT-negative isolates (*P* < 0.001) or uninfected controls (*P* = 0.003). This effect was consistent for all isolates tested (see Fig. S1 in the supplemental material). Specifically, 27.1% to 91.6% of HeLa cells infected with S-CDT-positive isolates representing serotypes Javiana, Montevideo, Mississippi, and Oranienburg showed both 53BP1 and γH2AX foci (double-positive cells) than 0.0% to 9.3% of HeLa cells infected with isolates representing S-CDT-negative serotypes. For HeLa cells infected with isolates representing S-CDT-negative serotypes, the proportion of double-positive cells also did not differ from that of uninfected controls (*P* = 0.992). As 53BP1 and γH2AX foci have been shown previously to localize to sites of DNA damage, these results also support the hypothesis that HeLa cells infected with S-CDT-positive serotypes sustain more DNA damage than HeLa cells infected with S-CDT-negative serotypes (29). Therefore, these results suggest that infection with isolates representing different S-CDT-encoding NTS serotypes results in DNA damage which activates the host cell DDR but that infection with isolates representing S-CDT-negative serotypes does not.

**FIG 4:**
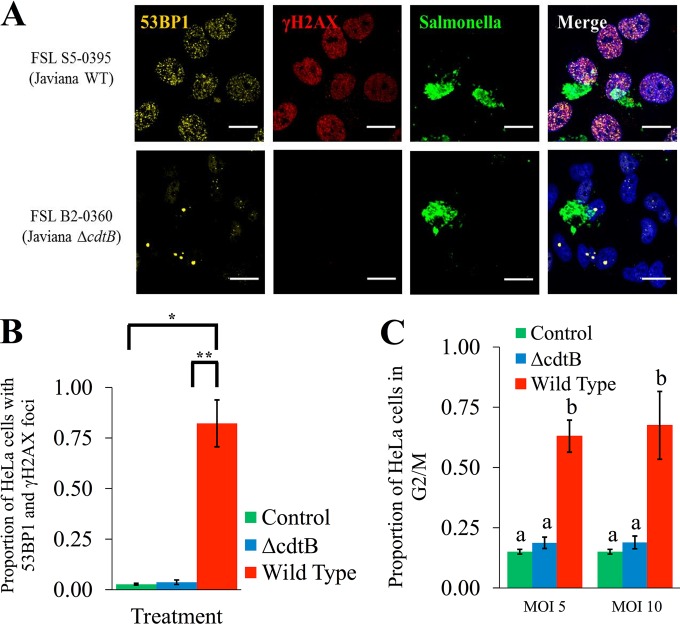
*cdtB* is essential for activation of the host DNA damage response and G_2_/M cell cycle arrest. HeLa cells infected with wild-type strains and Δ*cdtB* isogenic mutants of serotypes Javiana and Montevideo were analyzed for the presence of 53BP1 and γH2AX foci at 48 hpi. (A) Representative images showing 53BP1 and γH2AX foci among HeLa cells infected with a wild-type serotype Javiana strain and a Δ*cdtB* isogenic mutant. Scale bars, 25 μm. (B) Quantification of the proportion of HeLa cells with both 53BP1 and γH2AX foci; data represent the results for 2 technical replicates from 2 independent experiments for each of the 4 strains (Javiana and Montevideo parent strains and their respective isogenic mutants); error bars represent standard deviations. **, *P* value < 0.01; *, *P* value < 0.05. (C) Proportion of HeLa cell populations in the G_2_/M phase determined by DNA content analysis using flow cytometry. HeLa cells were infected at 2 multiplicities of infection with either wild-type strains or Δ*cdtB* isogenic mutants of serotypes Javiana and Montevideo. Cells were fixed at 48 hpi, and DNA content was measured using PI staining. Data represent the averages for both serotypes from 4 independent experiments for each strain. Bars that do not have identical letters represent statistically different values (*P* < 0.05). Error bars represent standard deviations.

To confirm that the observed activation of the host cell DDR was due to CdtB, we infected HeLa cells with Δ*cdtB* isogenic mutants of serotype Javiana and Montevideo isolates. Deletion of *cdtB* abolished the ability of these strains to activate the host cell DDR, as the proportion of Δ*cdtB* mutant-infected HeLa cells that were doubly positive for 53BP1 and γH2AX foci was not significantly different from those of uninfected controls (*P* = 0.805). HeLa cell populations infected with wild-type (WT) isolates of serotypes Javiana and Montevideo also had significantly (*P* = 0.004) higher proportions of cells with an activated DDR (i.e., with 53BP1 and γH2AX foci) than HeLa cell populations infected with the Δ*cdtB* isogenic mutants (Fig. 4A and B); immunofluorescence images indicated similar levels of intracellular *Salmonella* cells in HeLa cells infected with WT parent strains and their respective Δ*cdtB* isogenic mutants, suggesting that *cdtB* did not influence rates of invasion or intracellular survival within HeLa cells. Taken together, these results suggest that CdtB is essential for the activation of the DDR among cells infected with S-CDT-positive NTS serotypes.

### *cdtB* is required for the G_2_/M cell cycle arrest associated with infection with S-CDT-positive serotypes.

To confirm that the G_2_/M arrest was due to CdtB, we compared the cell cycle progressions of populations of HeLa cells infected with the WT Javiana and Montevideo strains and their Δ*cdtB* isogenic mutants. HeLa cells infected with the WT Javiana and Montevideo strains had a significantly higher proportion of cells in the G_2_/M phase at both an MOI of 5 (*P* < 0.001) and an MOI of 10 (*P* < 0.001) than cell populations infected with Δ*cdtB* isogenic mutants (Fig. 4C). There was no statistically significant effect of the MOI (5 or 10) on the proportion of HeLa cells that arrested in G_2_/M after infection with S-CDT-positive serotypes (*P* = 0.831), indicating that the MOI did not have a significant effect on the observed G_2_/M cell cycle arrest, even though immunofluorescence imaging showed that only a small proportion of HeLa cells were infected with *Salmonella* (Fig. S1) (at an MOI of approximately 5, only a median of 10% of HeLa cells were infected). Importantly, for either an MOI of 5 (*P* = 0.167) or an MOI of 10 (*P* = 0.149), the proportion of HeLa cells in the G_2_/M phase following infection with Δ*cdtB Salmonella* isolates was not significantly different from the proportion of uninfected control cells in the G_2_/M phase. Deletion of *cdtB* thus abolished the ability of the S-CDT-positive serotypes Javiana and Montevideo to arrest HeLa cells in the G_2_/M phase (Fig. 4C), demonstrating that CdtB is required for the G_2_/M cell cycle arrest associated with infection involving S-CDT-positive serotypes.

### S-CDT-mediated intoxication can occur via autocrine or paracrine pathways.

**FIG 5:**
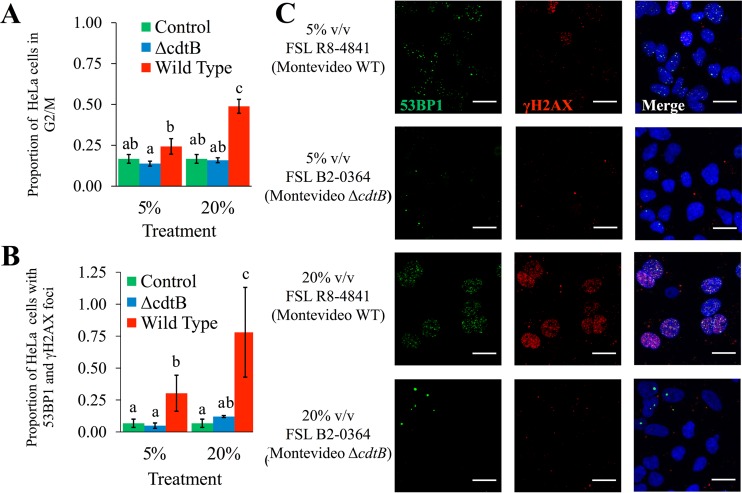
S-CDT-mediated activation of the DNA damage response and G_2_/M cell cycle arrest can occur via paracrine pathways. HeLa cells were coincubated with cell-free supernatants from previous infections with S-CDT-positive wild-type strains FSL S5-0395 (serotype Javiana) and FSL R8-4841 (serotype Montevideo) and their respective isogenic Δ*cdtB* mutants; supernatants were added at final concentrations of 5% and 20%, vol/vol (v/v). (A) At 24 h post-intoxication, cells were stained with PI to determine DNA content and to quantify the proportion of cells in the G_2_/M phase. Results represent data from 2 independent experiments for each of the 4 strains. Bars that do not have identical letters represent statistically different values (*P* < 0.05). (B) HeLa cells grown on coverslips were coincubated with cell-free supernatants from previous infections. Immunofluorescence staining was performed to detect cells with 53BP1 and γH2AX foci at 24 h post-intoxication. Bar charts represent data from 2 independent experiments. Bars that do not have identical letters represent statistically different values (*P* < 0.05). (C) Representative images are shown for supernatants from infections with FSL R8-4841 (WT strain of serotype Montevideo) and FSL B2-0364 (Δ*cdtB* mutant of serotype Montevideo). Data for FSL S5-0395 (Javiana WT) and FSL B2-0360 (Δ*cdtB* mutant of serotype Javiana) are shown in Fig. S1. Scale bars, 25 μm.

Immunofluorescence staining clearly showed that cells which were not infected with *Salmonella* but were located near *Salmonella*-infected HeLa cells had an activated DDR (Fig. 3A, 4A, and S1). To determine whether the activation of the DDR and G_2_/M arrest occur via intoxication arising from paracrine pathways, we coincubated uninfected HeLa cell populations with cell-free supernatants collected from previous infections with S-CDT-positive serotypes and their respective Δ*cdtB* isogenic mutants. HeLa cell populations incubated with cell-free supernatants from infections with WT isolates encoding S-CDT had a significantly higher proportion of cells in the G_2_/M phase than HeLa cells incubated with cell-free supernatants from Δ*cdtB* mutant-infected HeLa cells after incubation at total volumes of 5% (*P* = 0.034) and 20% (*P* = 0.014) (Fig. 5A). In contrast, the proportions of HeLa cells in the G_2_/M phase did not differ significantly between untreated cells and cells coincubated with either a 5% or a 20% (vol/vol) cell-free supernatant from a previous infection with Δ*cdtB* isogenic mutants (*P* = 0.450 and *P* = 0.979 for 5% and 20% [vol/vol] treatments, respectively).

We also found that HeLa cells coincubated with filter-sterilized cell-free supernatants from past infections with S-CDT-positive WT strains showed a significantly higher proportion of cells with 53BP1 and γH2AX foci than untreated control cells; this was observed at both concentrations of 5% (vol/vol) (*P* = 0.047) and 20% (vol/vol) (*P* = 0.004) (Fig. 5B and C). Furthermore, HeLa cells treated with cell-free supernatant from WT infections had a significantly higher proportion of cells having both 53BP1 and γH2AX foci than cells treated with cell-free supernatant from Δ*cdtB* mutant infections (*P* = 0.015 and *P* = 0.003 for 5% [vol/vol] and 20% [vol/vol] treatments, respectively). HeLa cells coincubated with cell-free supernatants from infections with Δ*cdtB* isolates also did not differ significantly from untreated control cells with regard to the proportion of cells with 53BP1 and γH2AX foci (*P* = 0.958 for 5% [vol/vol] treatment, *P* = 0.555 for 20% [vol/vol] treatment). Heat inactivation of supernatants collected from infection with S-CDT-positive isolates abolished the ability of the toxin-containing supernatant to induce 53BP1 and γH2AX foci (Fig. S2), suggesting that a heat-labile toxin is responsible for activation of the DDR. This is consistent with the results of previous studies which have shown that heating CDT-containing supernatants from *E. coli* and *Campylobacter* spp. at 70°C for 1 h abolishes the cytotoxic effect of this toxin in tissue culture (30, 31).

Previous studies have suggested a strict requirement for internalization of *Salmonella* cells in order for S-CDT production to occur (2, 16). Similarly, we found that coincubation of HeLa cells with either lysogeny broth (LB) or Eagle’s minimal essential medium (EMEM) used to culture S-CDT-positive *Salmonella* failed to induce activation of the host DDR and failed to induce a G_2_/M arrest (Fig. S3). This result suggests that the S-CDT production occurs only in intracellular *Salmonella* cells; hence, the paracrine route of S-CDT-induced intoxication requires initial intracellular infection of at least some host cells to allow for production of the S-CDT needed for the paracrine intoxication. Overall, these results suggest that intoxication with S-CDT may occur via autocrine or paracrine pathways, indicating that eukaryotic cells do not need to be infected with S-CDT-positive NTS in order to sustain DNA damage.

## DISCUSSION

Strains of nontyphoidal *Salmonella* (NTS) serotypes represent some of the most common causes of food-borne illnesses worldwide and therefore have a tremendous economic and public health impact. While most NTS infections cause mild gastrointestinal symptoms, systemic infections are not uncommon and may be associated with specific serotypes, such as serotype Dublin or Choleraesuis (19). In addition, long-term sequelae of NTS infections (e.g., reactive arthritis and postinfectious irritable bowel syndrome) have been reported (32). While recent studies of S-CDT production by S. Typhi have suggested that this virulence factor plays an important role in establishing a chronic infection and in the development of typhoid fever (8, 12), extensive genomic and phenotypic analyses have largely failed to definitively identify specific virulence factors that may be responsible for virulence differences among NTS serotypes, suggesting that multiple virulence factors likely contribute to the severity of an infection (20, 33). The recent identification of genes encoding S-CDT in the genomes of some NTS serotypes, however, has provided a specific set of genes that may impart unique virulence characteristics to a subset of NTS serotypes. As the specific contributions of S-CDT to the pathogenicity of NTS serotypes have remained largely uncharacterized, we conducted a series of experiments to assess the distribution and conservation of S-CDT-encoding genes among NTS serotypes and to determine the contributions of these genes to *Salmonella* pathogenicity using a cell culture model.

In this study, we established that among 21 NTS serotypes causing the majority of human clinical cases of salmonellosis in the United States, serotypes Javiana, Montevideo, and Oranienburg, as well as serotype Mississippi (some isolates), encode S-CDT. Interestingly, we found that serotype Mississippi is polyphyletic and that S-CDT-encoding genes are present only in isolates in 1 of the 2 clades. We estimated that infections with S-CDT-positive isolates representing these serotypes account for approximately 150,280 cases (90% CI, 111,812 to 196,954) of food-borne salmonellosis in the United States in 2011, suggesting that a relatively large population of individuals have been exposed to this toxin (17). In general, the results of our PCR screen agree with previous reports on the distribution of S-CDT-encoding genes among strains of NTS serotypes (20, 22–24). For example, using a much smaller set of isolates (1 to 2 isolates) per serotype, den Bakker et al. also reported that strains of serotypes Javiana, Montevideo, and Oranienburg carry *cdtB* (22). While our study focused on characterizing S-CDT’s presence among the most common NTS serotypes causing human salmonellosis in the United States, other studies indicated that in addition to serotypes Javiana, Montevideo, Oranienburg, and Mississippi, at least 37 other, rarer, NTS serotypes also encode S-CDT, suggesting further human exposure to S-CDT-positive NTS strains (22, 25). While some evidence supports the suggestion that S-CDT-positive NTS strains represent a specific NTS clade, there is at least some evidence that S-CDT is not exclusively associated with a specific clade (22). For example, a recent study by den Bakker et al. noted that *Salmonella* serotypes encoding S-CDT are found primarily in clade B (e.g., serotypes Javiana, Schwarzengrund, and Montevideo) but that a small subclade (including serotypes Paratyphi A and Typhi) of clade A also include strains that encode S-CDT (22). While future additional studies on the presence of S-CDT among additional NTS serotypes will be valuable, the data presented here on S-CDT’s presence among a large number of isolates representing serotypes commonly associated with human disease provide important information that allows for an initial assessment of the population in the United States that is exposed to S-CDT through infections involving NTS serotypes.

Not surprisingly, alignments of predicted amino acid sequences suggested that CdtB, PltA, and PltB are highly conserved across serotypes, with the active subunit (CdtB) having the fewest amino acid substitutions. It is interesting to note that several single-nucleotide polymorphisms (SNPs) in the coding sequences of PltB and PltA involved transitions to codons which are less frequently used (i.e., “rare”) by *Salmonella*, potentially leading to a reduction in the overall quantities of these proteins in some strains (34). Importantly, our data suggest that the S-CDT produced by NTS serotypes, based on overall nucleotide similarity to coding sequences of S-CDT genes carried by S. Typhi likely induces DNA damage in eukaryotic cells in a manner similar to that of the S-CDT produced by S. Typhi.

To date, few studies have specifically examined the contributions of S-CDT production by NTS serotypes to infection and pathogenesis (23–25). While limited prior studies have shown that some S-CDT-positive serotypes (e.g., Javiana) induce a G_2_/M arrest (23, 25), our results not only confirm that infection with S-CDT-positive NTS serotypes causes a cell cycle arrest that is not observed in cells infected with S-CDT-negative serotypes but also show that this cell cycle arrest is associated with activation of the host DDR. In addition, we show that serotypes Oranienburg and Mississippi induce DNA damage in infected eukaryotic cells, further confirming that an active S-CDT is produced by these NTS serotypes and that the DNA-damaging effects are not restricted to serotype Typhi. Our data show that infection with wild-type strains of S-CDT-positive NTS serotypes significantly impacts the cellular outcome of infection, suggesting that S-CDT is an important pathogenicity factor in strains that encode S-CDT.

An important difference between S-CDT and the CDTs produced by other Gram-negative pathogens is the additional ADP-ribosyl transferase activity of the PltA subunit in S-CDT (2, 3). Here, we confirmed that the DNA-damaging effects are due to the activity of the CdtB subunit, as deletion of *cdtB* abolished the ability of S-CDT-positive serotypes to cause DNA damage (as shown by the G_2_/M cell cycle arrest and the 53BP1 and γH2AX foci). This is in agreement with the findings of previous studies of serotype Typhi which also showed that *cdtB* deletion abolishes G_2_/M cell cycle arrest (2, 16). We therefore conclude that the S-CDT produced by NTS acts in the same manner as the S-CDT produced by serotype Typhi.

Immunofluorescence staining of HeLa cells infected with S-CDT-positive NTS isolates showed that the DNA damage sustained from S-CDT-mediated intoxication is not restricted to infected cells but impacts nearby cells as well. Previous studies with S. Typhi have similarly shown that intoxication with S-CDT may occur via autocrine or paracrine pathways, as intoxication of Henle cells in the presence of a toxin-neutralizing antibody abolished the cell cycle arrest of cells coincubated with cell-free supernatants containing S-CDT from previous infections (2). In support of this, a previous study also showed that exogenous addition of purified S-CDT was sufficient to cause a G_2_/M-phase arrest, suggesting that S-CDT is necessary and sufficient for the cytotoxic activity observed in S-CDT-encoding serotypes of *Salmonella* (12). Furthermore, Guidi et al. showed that S-CDT is transported anterograde along host microtubules of infected cells in outer membrane vesicles and secreted from infected cells into the surrounding media (35). Consequently, it is likely that all cells, whether or not they are infected with *Salmonella*, sustain S-CDT-mediated DNA damage via the same mechanism, as all cells appear to take up S-CDT from the extracellular environment. This is important because during an infection, relatively few *S. enterica* cells successfully invade host cells (36). Together, these results suggest that dissemination of S-CDT into the surrounding tissues has the potential to impact a greater number of host cells, which may contribute to the overall outcome of infection.

It has been established previously that *Salmonella* may induce host cell death via multiple mechanisms, including apoptosis and pyroptosis, depending on the host cell type (e.g., immune versus epithelial cells) (37–39). The majority of these analyses have been conducted using serotype Typhimurium, which is now known to be S-CDT negative. Therefore, S-CDT-mediated intoxication represents an important new mechanism by which NTS may induce cell death. While HeLa cells represent a standard cell line used for studies of DNA damage, future studies examining the cellular outcome of infection with S-CDT-positive NTS serotypes in noncancerous (i.e., nontransformed) cell lines will be important to assess whether eukaryotic cells are able to repair the DNA damage induced by S-CDT-positive NTS serotypes.

Our data indicate that S-CDT significantly impacts the outcome of infection with NTS serotypes at the cellular level. It will be important to further assess the role of S-CDT *in vivo* to determine the implications of S-CDT-mediated DNA damage at the host level and to also ascertain what, if any, long-term sequelae may result from the DNA damage sustained during an infection with S-CDT-producing NTS. An analysis by Rodriguez-Rivera et al. (25) reported that nontyphoidal serotypes which had a higher proportion of infections resulting in invasive disease (as reported by Jones et al. [19]) were significantly more likely to encode S-CDT, therefore suggesting that S-CDT contributes to the severity of human clinical cases as well (19, 25). AbuOun et al. (40) found that sera from humans who had previously been exposed to *Campylobacter jejuni* (which encodes a CDT) neutralized the activity of the toxin *in vitro*, suggesting that individuals seroconvert following exposure to CDTs (40). Further studies will be beneficial in determining whether S-CDT exposure also causes antibody production and potentially the neutralization of S-CDT produced by NTS.

Overall, the data provided here contribute important new information that strongly suggests that a subset of NTS strains (i.e., those encoding S-CDT) are likely to cause unique consequences of infection at both the cellular and the host level. Hence, there is an urgent need to determine the potential public health impacts of S-CDT-producing NTS serotypes. Ultimately, this work may lead to differential assessment of the food safety hazards posed by foods contaminated with S-CDT-positive and -negative NTS strains.

## MATERIALS AND METHODS

### Bacterial strains, human cell lines, and culturing conditions.

A total of 864 wild-type *Salmonella* isolates obtained from foods (e.g., raw oysters, shrimp, jalapeño peppers, spices, and others), environmental samples (e.g., ground water, soil, drag swabs from produce fields, and dairy and avian farm environments), animals (e.g., bovines, avians, and reptiles), and humans with clinical symptoms in the United States were selected from an existing strain collection for inclusion in this study (Table 3; see also Data Set S1 in the supplemental material for a complete list). Isolates were selected to belong to 21 NTS serotypes most frequently isolated from human clinical cases of salmonellosis in the United States in 2011, as reported by the 2011 CDC National Salmonella Surveillance report (47). Approximately 50 isolates were selected for each serotype, except for 7 serotypes (Table 1), for which only a small number of isolates could be obtained from the Cornell Food Safety Laboratory’s *Salmonella* collection. In addition, the isogenic Δ*cdtB* mutants FSL B2-0360 and FSL B2-0364 and the corresponding parent strains FSL S5-0395 (serotype Javiana) and FSL R8-4181 (serotype Montevideo) were used (Table 3); these strains, including their Δ*cdtB* mutants, had previously been described (25). As previously detailed, Δ*cdtB* mutants were constructed by deleting the entire *cdtB* open reading frame (ORF) using the λ-Red recombinase method (41). All strains were maintained in 15% (vol/vol) glycerol stored at −80°C. *Salmonella* cells were grown on brain heart infusion (BHI; Becton, Dickinson, Sparks, MD) agar plates, which were incubated at 37°C. For infections, *Salmonella* strains were cultured in LB (pH 8; 0.3 M NaCl) under static conditions at 37°C to increase invasion efficiency (42–44).

**TABLE 3:** *Salmonella* strains used for *in vitro* analyses

Strain	Serotype	Genotype	Source	Date isolated^a^
FSL S5-0415	Enteritidis	Wild type	Human	April 2004
FSL S5-0536	Typhimurium	Wild type	Human	November 2004
FSL S5-0639	Newport	Wild type	Human	December 2004
FSL S5-0395	Javiana	Wild type	Human	March 2004
FSL R8-2779	Mississippi	Wild type	Human	November 2008
FSL R8-4841	Montevideo	Wild type	Human	April 2010
FSL S5-0642	Oranienburg	Wild type	Human	December 2004
FSL B2-0360^b^	Javiana	FSL S5-0395 Δ*cdtB*	Lab strain	NA
FSL B2-0364^b^	Montevideo	FSL R8-4841 Δ*cdtB*	Lab strain	NA

a NA, not applicable.

b This mutant and its construction were previously described (25).

HeLa cells were grown in Eagle’s minimum essential medium (EMEM; Gibco-Invitrogen, Carlsbad, CA) supplemented with 10% (vol/vol) fetal bovine serum (FBS; Gibco-Invitrogen) and were incubated at 37°C with 5% CO_2_. For infection, HeLa cells were grown in 6-well or 12-well plates (Corning, Corning, NY) to the desired confluence (40 to 50% confluence for flow cytometry analyses or 40 to 70% confluence for immunofluorescence analyses).

### PCR screen for S-CDT genes *pltA*, *pltB*, and *cdtB*.

Isolates were plated on BHI agar plates from frozen glycerol stocks, followed by overnight incubation at 37°C. Cell lysates were prepared by inoculating 100 μl of sterile water with bacterial cells, followed by heating them at 95°C for 15 min in a thermocycler. Each isolate was screened for genes encoding the 3 components of S-CDT (*pltA*, *pltB*, and *cdtB*) using the primers and their respective annealing temperatures listed in Table S1. Screening was performed using 2 PCRs. The first PCR mixture contained primers to amplify (i) a portion of the 16S rRNA gene (included as a positive control) and (ii) an internal fragment of *pltA*. The second PCR was used to simultaneously amplify internal regions of *pltB* and *cdtB*. Thermocycling conditions included an initial denaturation at 94°C for 3 min, followed by 30 cycles of denaturation at 94°C for 30 s, annealing at either 48°C or 50°C (Table S1) for 30 s, and extension at 72°C for 1 min; a final extension at 72°C for 7 min was also included. PCR products were visualized using agarose gel electrophoresis. In the event that PCR amplification did not yield amplicons for the 16S rRNA gene, a new lysate was prepared and both PCRs were performed again.

### Sequencing of full-length *pltA*, *pltB*, and *cdtB* for phylogenetic comparison.

Full-length gene segments were amplified for 5 representative isolates (with the exception of those of serotype Mississippi, only 4 of which were S-CDT positive) from each serotype or clade that was identified as S-CDT positive. Primers were designed to anneal to regions upstream and downstream of *pltA*, *pltB*, and *cdtB* (Table S1). PCR amplification was performed using a touchdown method (for *pltA* and *pltB*), with initial denaturation at 94°C for 3 min, followed by 20 cycles of denaturation at 94°C for 30 s, with annealing temperature decreasing by 0.5°C per cycle (from 60°C at cycle 1 to 50°C at cycle 20) (Table S1) and extension at 72°C for 1.5 min; an additional 20 cycles of amplification were then performed using an annealing temperature of 50°C, with a final extension at 72°C for 7 min. For *cdtB*, amplification was performed using the following thermocycling conditions: 94°C for 3 min, followed by 30 cycles of denaturation at 94°C for 30 s, annealing at 50°C for 30 s, and elongation for 1 min at 72°C, followed by a final extension at 72°C for 5 min. PCR products were treated with 5 units of exonuclease I (Affymetrix, Santa Clara, CA) and 5 units of shrimp alkaline phosphatase (Affymetrix), followed by Sanger sequencing performed by the Cornell Biotechnical Resource Center (Cornell University, Ithaca, NY). Sequences were edited using Sequencher software (Gene Codes Corporation, Ann Arbor, MI). Phylogenetic analyses were performed using Mega software (version 6).

### Seven-gene MLSTs.

For selected isolates, multilocus sequence typing was performed as described previously, using internal fragments of the housekeeping genes *aroC*, *dnaN*, *hemD*, *hisD*, *purE*, *sucA*, and *thrA* (26). Sequences were edited and compared to those in the *S. enterica* MLST database, which allows for assignment of MLSTs (26).

### *Salmonella* infection of HeLa cells.

Bacterial cultures were plated from frozen glycerol stocks onto BHI agar plates, followed by incubation at 37°C for 22 to 24 h. Subsequently, single colonies were used to inoculate 5-ml aliquots of LB (pH 8; 0.3 M NaCl), followed by incubation under static conditions at 37°C for 14 ± 1 h. These cultures were then subcultured 1:100 into fresh 5-ml aliquots of LB (pH 8; 0.3 M NaCl), followed by incubation at 37°C under static conditions until bacteria reached early to mid-log phase (optical density at 600 nm [OD_600_] of 0.4 ± 0.1). Treatments (i.e., the level of inoculum and strain of NTS used) were randomly assigned to HeLa cells seeded in 6-well or 12-well plates. HeLa cells were infected with bacterial cultures at multiplicities of infection (MOIs) of approximately 5 and 10. After incubation of the infected cells for approximately 1 h at 37°C (with 5% CO_2_), HeLa cells were washed 3 times with phosphate-buffered saline (PBS), followed by incubation with EMEM supplemented with 100 μg/ml gentamicin (Gibco) for 1.25 h at 37°C to kill extracellular bacteria. Subsequently, HeLa cells were washed an additional 3 times with PBS and were then maintained in EMEM containing 10 μg/ml gentamicin (Gibco) to prevent recurrent infection and bacterial outgrowth during incubation.

### Flow cytometry.

After the selected incubation periods, HeLa cells were washed once with PBS and were harvested using 0.5% trypsin-EDTA (Gibco). Cells were fixed in 70% ethanol at −20°C for at least 3 h. Ethanol-fixed cells were permeabilized with PBS containing 0.1% Tween 20 (PBS-T) and bovine serum albumin (1 g/100 ml) (Sigma-Aldrich, St. Louis, MO) at room temperature for 10 min. Cells were subsequently stained (10 min at room temperature) with a solution containing propidium iodide (PI) (Thermo Fisher Scientific, Waltham, MA) at a final concentration of 50 μg/ml and RNase A (Sigma-Aldrich) at a final concentration of 100 μg/ml. Stained cells were held at 4°C for no longer than 4 h, prior to DNA content analysis using the BD FACSAria. Cells were gated to exclude doublets and multiplets, as described previously (45).

### Immunofluorescence staining.

HeLa cells grown on 12-mm coverslips (Thermo Fisher Scientific) seeded in 12-well plates were infected with NTS strains at an MOI of approximately 5, as described above. At 48 h postinfection (hpi), cells were washed with PBS and then fixed with 4% paraformaldehyde in PBS at room temperature for 15 to 20 min. Slides were washed with PBS, followed by permeabilization with 0.5 to 1% Triton X-100 in PBS at room temperature for 10 min. Permeabilized cells were then blocked with 10% horse serum (Gibco) in PBS-T for 1 h at room temperature. Incubation with primary antibodies was performed for 1 h at room temperature using a dilution factor in PBS-T of 1:500 for polyclonal goat anti-*Salmonella* antibody (KPL, Gaithersburg, MA), mouse anti-γ-H2AX (EMD Millipore, Billerica, MA), and rabbit anti-53BP1 (Novus Biologicals, Inc., Littleton, CO). Incubation with secondary antibodies diluted 1:200, except where noted, in PBS-T was performed for 1 h at room temperature; antibodies used were donkey anti-mouse conjugated to Alexa 405 (Abcam, Inc., Cambridge, MA), donkey anti-goat conjugated to Alexa 488, donkey anti-rabbit conjugated to Alexa 555 (diluted 1:500), and donkey anti-mouse conjugated to Alexa 647 (all Thermo Fisher Scientific). Nuclei were stained with either 4′,6-diamidino-2-phenylindole (DAPI) at a final concentration of 1 μg/ml or PI at a final concentration of 40 μg/ml for 5 min at room temperature. Slides were subsequently mounted onto microscope slides with Glycergel (Dako, Carpinteria, CA) and were imaged using a Zeiss 710 confocal microscope. Two z-stacks (representing 2 independent fields of view) were collected and analyzed per slide. Images were processed with Fiji software, and cells were counted using the cell counter plug-in (46). At least 90 nuclei were analyzed per slide to identify cells that had more than four 53BP1-positive foci and were also positive for γH2AX; these cells were designated 53BP1- and γH2AX-positive cells (or doubly positive cells). The observer was blind to the treatment while collecting and analyzing images.

### Cell-free supernatant intoxication.

Medium supernatants were collected at 48 hpi from HeLa cells infected with different *Salmonella* strains and were filtered through a 0.2-µm filter to remove bacterial cells. The resulting cell-free supernatants were coincubated with fresh HeLa cell monolayers for 24 h. Following incubation, cells were fixed for flow cytometry or for immunofluorescence microscopy as described above. Cell-free supernatants were also tested after heat treatment (70°C or 95°C for 10 min).

### Statistical analysis.

All statistical analyses were performed using R software (version 3.0.2; R Project, Vienna, Austria). Linear mixed-effects models were used to assess significant associations between S-CDT status (the presence or absence of S-CDT-encoding genes or the WT versus Δ*cdtB* isogenic mutants) and either (i) proportions of cell populations in G_2_/M or (ii) proportions of cells with an activated DDR as determined using immunofluorescence staining for 53BP1 and γH2AX foci. The MOI and S-CDT status were included as fixed effects. To account for potential variability due to (i) the passage number of the HeLa cells and (ii) the *Salmonella* strain, these variables were included in models as random effects. An interaction term was included for S-CDT status and the MOI, when appropriate. Data were transformed by taking the log_10_, natural log, and square root or performing a rank transformation, when appropriate to obtain a normal distribution of the residuals. Data shown in figures represent untransformed data. The lsmeans (least-square means) package was used to perform pairwise comparisons among treatments, and *P* values were adjusted using the Tukey method to account for multiple comparisons. The 90% confidence interval for the binomial proportion of human Mississippi isolates positive for S-CDT was estimated using the Wilson score interval.

### Accession number(s).

Sequence data for all S-CDT subunits sequenced are available in NCBI’s GenBank (see Data Set S2 in the supplemental material for accession numbers) and in the publicly available online database http://www.foodmicrobetracker.com.

## SUPPLEMENTAL MATERIAL

Data Set S1  All *Salmonella* isolates screened for *pltA*, *pltB*, and *cdtB*. Download Data Set S1, XLSX file, 0.01 MB

Data Set S2  GenBank accession numbers for *pltA*, *pltB*, and *cdtB* sequences for *Salmonella* strains characterized in this study. Download Data Set S2, XLSX file, 0.05 MB

Figure S1  Infection with S-CDT-positive serotypes activates the host DNA damage response, while infection with S-CDT-negative serotypes does not. HeLa cells were infected with S-CDT-positive isolates, S-CDT-negative isolates, and Δ*cdtB* isogenic mutants at a multiplicity of infection of approximately 5. Immunofluorescence staining was performed to detect 53BP1 and γH2AX foci at 48 hpi. Representative images are included for all serotypes. Scale bars, 25 μm. Download Figure S1, TIF file, 53.7 MB

Figure S2  Heat treatment of supernatants from previous infections inactivates S-CDT-mediated activation of the DNA damage response. Supernatants were collected at 48 h postinfection from HeLa cells that were initially infected (or uninfected, in the case of the “uninfected control”) with *Salmonella*; the *S. enterica* serotype Enteritidis WT strain is S-CDT-negative, while the *S. enterica* serotype Javiana WT strain is S-CDT-positive. Supernatants were filtered with a 0.2-μm filter and were subsequently heat treated at 95°C for 10 min. These supernatants were then added (final volume, 10% [vol/vol]) to HeLa cell cultures and were incubated for 24 h prior to fixation with 4% paraformaldehyde (PFA). Immunofluorescence staining was performed to detect 53BP1 (green) and γH2AX (red) foci. Nuclei were stained with DAPI. Scale bars, 25 μm. Download Figure S2, TIF file, 40.3 MB

Figure S3  S-CDT-mediated intoxication does not occur when *Salmonella* cells are grown in LB or in EMEM. (A) *Salmonella* cells were cultured in 0.3 M NaCl LB, pH 8, at 37°C under stationary conditions until mid-log phase; the LB was filtered with a 0.2-μm filter to remove bacterial cells, and the resulting filtered broth (at a final concentration of 10% [vol/vol]) was added to HeLa cells grown on glass coverslips in 24-well plates. After 24 h, HeLa cells were fixed with 4% PFA, and immunofluorescence staining was performed to detect γH2AX (red) and 53BP1 (green) foci. DAPI is included as a nucleic acid stain. Uninoculated LB was included as a negative control, and 2 μM etoposide was included as a positive control. Scale bars, 25 μm. (B) HeLa cells grown in 6-well plates were coincubated with sterile-filtered LB or EMEM inoculated with S-CDT-positive *Salmonella* cells (wild-type *S. enterica* serotype Javiana FSL S5-0395) or S-CDT null *Salmonella* cells (*S. enterica* serotype Javiana FSL B2-0360 [Δ*cdtB*]) at a final concentration of 10% (vol/vol). After 24 h, cells were harvested and subjected to flow cytometry to determine cell cycle phase based on DNA content (i.e., G_1_, S, or G_2_/M cell cycle phase). Download Figure S3, TIF file, 40.3 MB

Table S1  Primers used in this study. Table S1, DOCX file, 0.02 MB
