# Supplementary figures and images for: The Cytolethal Distending Toxin Produced by Nontyphoidal Salmonella Serotypes Javiana, Montevideo, Oranienburg, and Mississippi Induces DNA Damage in a Manner Similar to That of Serotype Typhi
Source: mBio. 2016 Dec 20;7(6):e02109-16. doi: 10.1128/mBio.02109-16 (PMC5181781; doi:10.1128/mBio.02109-16)

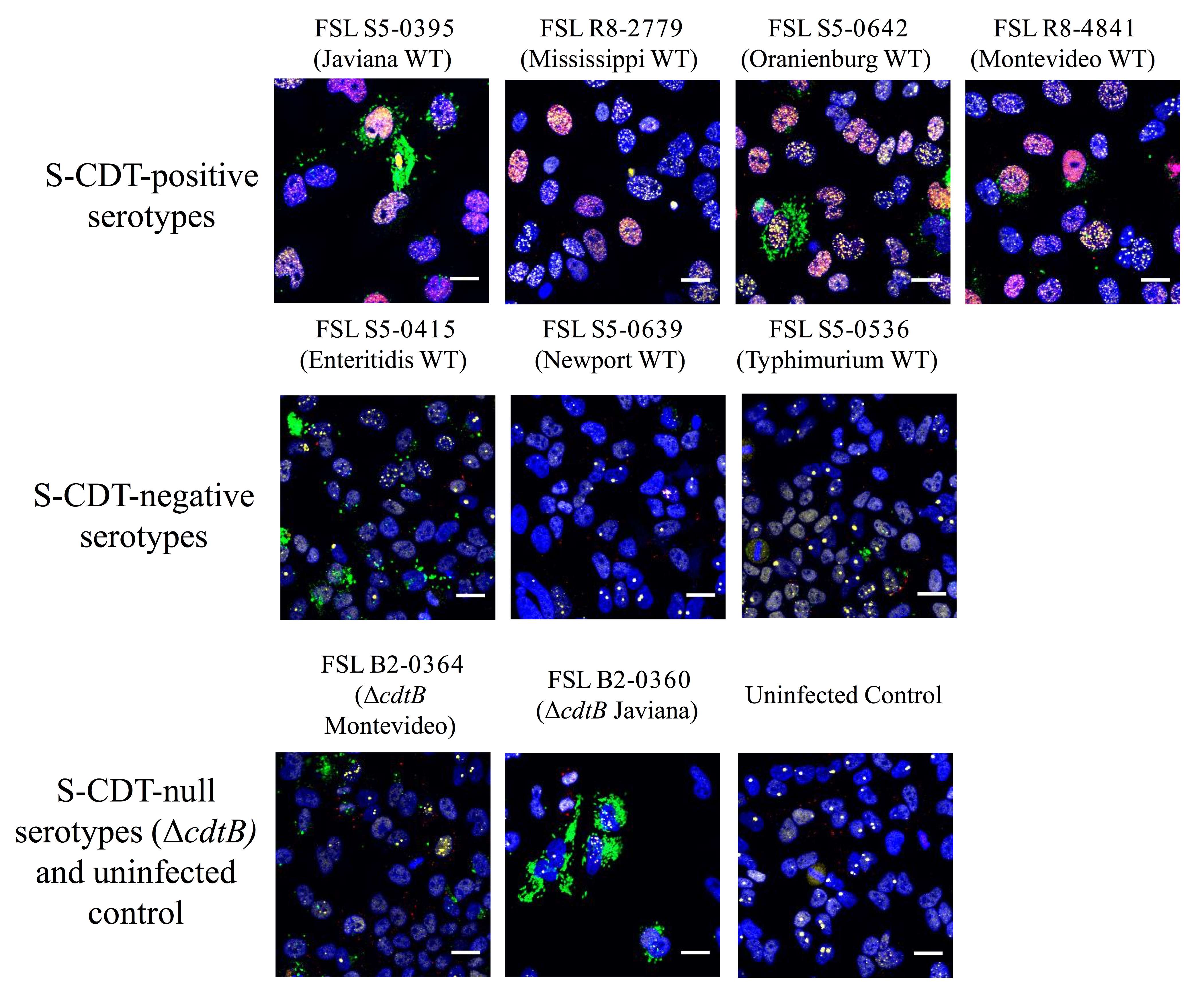

Supplement: Figure S1 — Infection with S-CDT-positive serotypes activates the host DNA damage response, while infection with S-CDT-negative serotypes does not. HeLa cells were infected with S-CDT-positive isolates, S-CDT-negative isolates, and ΔcdtB isogenic mutants at a multiplicity of infection of approximately 5. Immunofluorescence staining was performed to detect 53BP1 and γH2AX foci at 48 hpi. Representative images are included for all serotypes. Scale bars, 25 μm. Download [file mbo006163116sf1.tif]

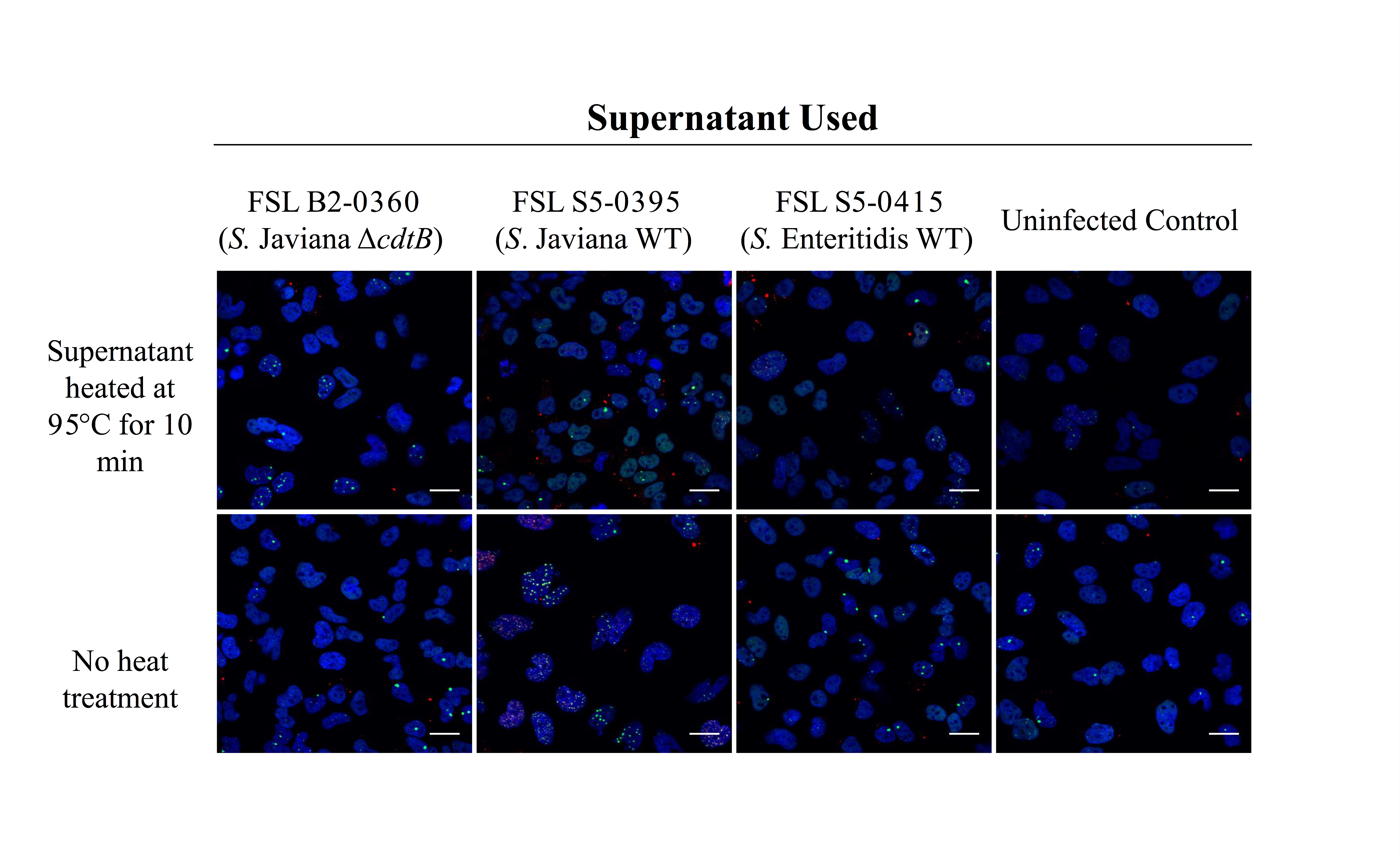

Supplement: Figure S2 — Heat treatment of supernatants from previous infections inactivates S-CDT-mediated activation of the DNA damage response. Supernatants were collected at 48 h postinfection from HeLa cells that were initially infected (or uninfected, in the case of the “uninfected control”) with Salmonella; the S. enterica serotype Enteritidis WT strain is S-CDT-negative, while the S. enterica serotype Javiana WT strain is S-CDT-positive. Supernatants were filtered with a 0.2-μm filter and were subsequently heat treated at 95°C for 10 min. These supernatants were then added (final volume, 10% [vol/vol]) to HeLa cell cultures and were incubated for 24 h prior to fixation with 4% paraformaldehyde (PFA). Immunofluorescence staining was performed to detect 53BP1 (green) and γH2AX (red) foci. Nuclei were stained with DAPI. Scale bars, 25 μm. Download [file mbo006163116sf2.tif]

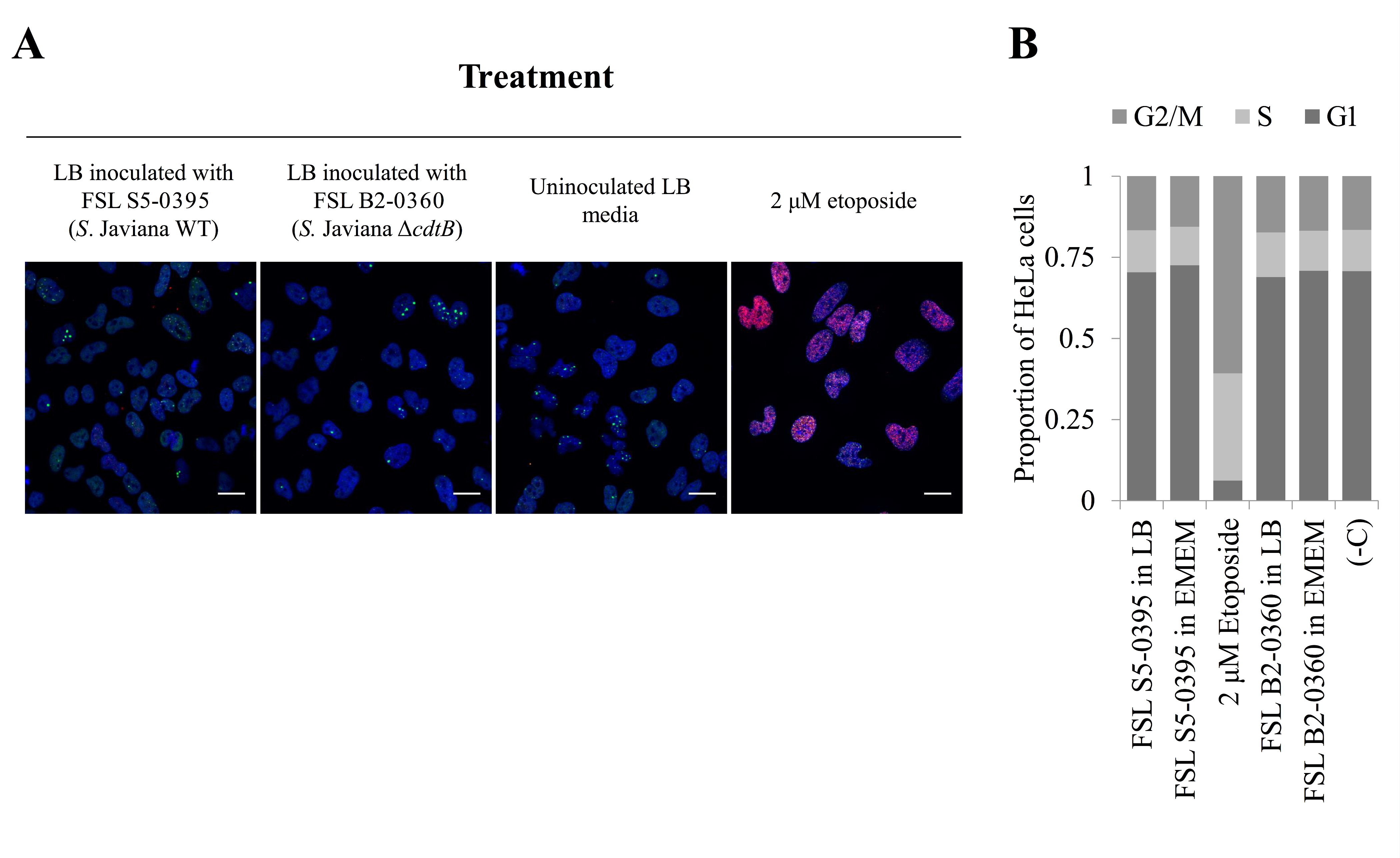

Supplement: Figure S3 — S-CDT-mediated intoxication does not occur when Salmonella cells are grown in LB or in EMEM. (A) Salmonella cells were cultured in 0.3 M NaCl LB, pH 8, at 37°C under stationary conditions until mid-log phase; the LB was filtered with a 0.2-μm filter to remove bacterial cells, and the resulting filtered broth (at a final concentration of 10% [vol/vol]) was added to HeLa cells grown on glass coverslips in 24-well plates. After 24 h, HeLa cells were fixed with 4% PFA, and immunofluorescence staining was performed to detect γH2AX (red) and 53BP1 (green) foci. DAPI is included as a nucleic acid stain. Uninoculated LB was included as a negative control, and 2 μM etoposide was included as a positive control. Scale bars, 25 μm. (B) HeLa cells grown in 6-well plates were coincubated with sterile-filtered LB or EMEM inoculated with S-CDT-positive Salmonella cells (wild-type S. enterica serotype Javiana FSL S5-0395) or S-CDT null Salmonella cells (S. enterica serotype Javiana FSL B2-0360 [ΔcdtB]) at a final concentration of 10% (vol/vol). After 24 h, cells were harvested and subjected to flow cytometry to determine cell cycle phase based on DNA content (i.e., G1, S, or G2/M cell cycle phase). Download [file mbo006163116sf3.tif]
