# Supplementary material for: The Cytolethal Distending Toxin Produced by Nontyphoidal Salmonella Serotypes Javiana, Montevideo, Oranienburg, and Mississippi Induces DNA Damage in a Manner Similar to That of Serotype Typhi
Source: mBio. 2016 Dec 20;7(6):e02109-16. doi: 10.1128/mBio.02109-16 (PMC5181781; doi:10.1128/mBio.02109-16)
Supplement: Table S1 — Primers used in this study. [file mbo006163116st1.docx]

**Table S1**. Primers used in this study

| **Primer Name** | **Primer Sequence (5' to 3')** | **Annealing Temperature^a^** | **Expected Amplicon Size (bp)** |
| --- | --- | --- | --- |
| RM45cdtBintF_2 | CTGCGCTAATATCAGTGACTAC | 48°C | 639 |
| RM46cdtBtintR_2 | CAACCCTTTGTGAATAAGGTGC |  |  |
| RM47pltBintF_2 | ACGATGATAGTCCCACAAGAGC | 48°C | 243 |
| RM48pltBintR_2 | CCTACTACTCAGACGAAGTTATC |  |  |
| RM49pltAintF_2 | ATCACGCCAGGATTAGACGTAG | 50°C | 313 |
| RM50pltAintR_2 | GCTATATTGCAACAACCTCAAGTG |  |  |
| 16s-PEU7 | GCAAACAGGATTAGATACCC | 50°C | 700 |
| 16s-P3SH | CTACGGTTACCTTGTTACGACTT |  |  |
| RM23PltAupstream | CCAGACCACTAATAAACGGTCTG | 60-50°C | 801 |
| RM24PltAdownstream | TATCATCGTGCAGTGCGA |  |  |
| RM25PltBupstream | GACTGGACTGTGATGTGGTG | 60-50°C | 545 |
| RM26PltBdownstream | GTTGAGTCCACACGATACAC |  |  |
| RM97cdtBupF | CAACGTCATGAAACAATGGGTTATG | 57°C | 935 |
| RM96cdtBdownR | ATATTCTGCACCTTACGCTCAAAGTAC |  |  |

^a^Annealing temperature used in PCR amplification; annealing temperatures reported as a range were performed using a range of temperatures as a part of a touchdown PCR
